# Towards a Design Process for Computer-Aided Biomimetics

**DOI:** 10.3390/biomimetics3030014

**Published:** 2018-06-21

**Authors:** Ruben Kruiper, Julian F. V. Vincent, Eitan Abraham, Rupert C. Soar, Ioannis Konstas, Jessica Chen-Burger, Marc P. Y. Desmulliez

**Affiliations:** 1 Deparment of Mathematical and Computer Sciences, Heriot-Watt University, Edinburgh Campus, Edinburgh EH14 4AS, UK; i.konstas@hw.ac.uk (I.K.); y.j.chenburger@hw.ac.uk (J.C.-B.); 2 School of Engineering and Physical Sciences, Heriot-Watt University, Edinburgh Campus, Edinburgh EH14 4AS, UK; jv21@hw.ac.uk (J.F.V.V.); e.abraham@hw.ac.uk (E.A.); m.desmulliez@hw.ac.uk (M.P.Y.D.); 3 School of Architecture Design and the Built Environment, Nottingham Trent University, 50 Shakespeare St, Nottingham NG1 4FQ, UK; rupert.soar@ntu.ac.uk

**Keywords:** Computer-Aided Biomimetics (CAB), Biologically Inspired Design (BID), biomimicry, biomimetics, bionics, design theory, innovation, invention, problem-solving

## Abstract

Computer-Aided Biomimetics (CAB) tools aim to support the integration of relevant biological knowledge into biomimetic problem-solving processes. Specific steps of biomimetic processes that require support include the identification, selection and abstraction of relevant biological analogies. Existing CAB tools usually aim to support these steps by describing biological systems in terms of functions, although engineering functions do not map naturally to biological functions. Consequentially, the resulting static, functional view provides an incomplete understanding of biological processes, which are dynamic, cyclic and self-organizing. This paper proposes an alternative approach that revolves around the concept of trade-offs. The aim is to include the biological context, such as environmental characteristics, that may provide information crucial to the transfer of biological information to an engineering application. The proposed design process is exemplified by an illustrative case study.

## 1. Introduction

This article focuses on finding solutions for technical problems that are inspired by nature. To this end, the definition for biomimetics by Fayemi et al. is followed, namely “the interdisciplinary creative process between biology and technology, aiming to solve technospheric problems through abstraction, transfer and application of knowledge from biological models” [1]. Unlike in engineering, functionalities encountered in nature are often hierarchical, dynamical and rely on information embedded at various hierarchical levels. As a result, biomimetics remains adventitious and is not used as widely and often as it potentially could be [2]. Problem-driven biomimetics processes are scarcely automated and usually take between 6 and 18 months to get from a specific problem to a functional prototype [3,4]. To enable a more systematic application of biomimetics, computational tools are required that integrate large amounts of biological knowledge in a given framework amenable to a methodology suitable for engineering.

The initial aim of this article is to elucidate the requirements for such computational tools:

- Avoid a bias towards engineering terminology and engineering functions, in order to preserve contextual information that is present in biological terminology; see Section 3.1 and Section 3.2.
- Extract structured, within-domain information from biology research papers to enable reliable information retrieval; see Section 3.3.
- Avoid subsequent automated mapping between the biological and engineering domains, taking into consideration the semantic distance between these domains; see Section 3.3 and Section 3.4.
- Support the direct and indirect uses of various theoretical models for biomimetics to represent the information found in biological texts, hence a model-agnostic tool; see Section 3.4.

Section 2 addresses the support that Computer-Aided Biomimetics (CAB) tools should provide following a literature review [1,5,6,7,8,9]. The search for biological information is thought to be most comprehensive and effective if biological texts are used [10]. Support for the identification and filtering of relevant texts is often provided by linking biological systems to other domains of knowledge through the concept of function. The notion of a function bridge between the biological and engineering domains is sometimes invoked [11]. However, as described in Section 3, the role of function in biomimetics is not always justified or appropriate, especially during the automated identification of relevant biological texts [10,12,13,14,15,16]. A reader who is aware of the limitations of a functional approach in CAB may skip Section 2 and Section 3. These sections are intended to provide a theoretical background for the high-level requirements listed above.

Finding a suitable bridge to transfer knowledge between the domains of biology and engineering is challenging and remains a current topic of research. The assumption is that a direct transfer is often not feasible. Instead multiple biological systems may inspire a compound solution; see Section 4 on iterative design [17,18]. Section 5 provides a brief overview of existing CAB tools [19,20,21]. Shortcomings of these systems include their bias towards engineering terminology and the partial omission of systemic context implied in biological terms.

The secondary aim of this article is to present progress towards an alternative approach to CAB that satisfies the requirements listed above. Section 6 introduces this approach in which the iterative search for relevant biological information is initially guided by a trade-off between two (or more) high-level features of a system. The purpose of a computational tool is to reduce the effort and time required to perform the proposed approach to biomimetics. In the proposed approach, this reduction is achieved by enabling a user to process more relevant raw biological information in a short amount of time. The task of a CAB tool that meets the requirements listed above is then to (1) present the important concepts and relations found verbatim in biological information sources and (2) improve retrieval by extracting trade-offs.

## 2. The Problem of Finding Relevant Biological Systems

**Figure 1:**
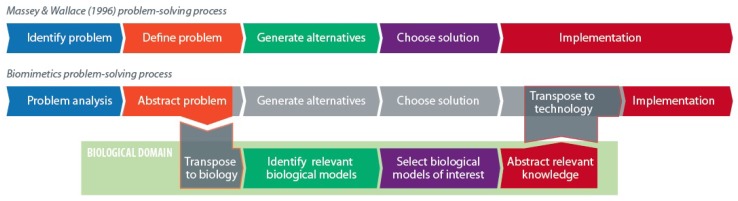
Overview of simplified, classical problem-solving (**top**) and generic biomimetics (**bottom**). In reality the designer has to know the external constraints in order to judge, e.g., whether transposing the selected biological information to technology is feasible and what the performance might be like [23]. Adapted from Fayemi et al. [1,5].

Figure 1 shows the difference between generic problem-solving as presented by Massey and Wallace, and taking inspiration from nature when solving a problem [22]. Both processes are depicted as a series of steps to get from a problem to a solution. Engineers who want to solve a biomimetic problem require support during the three steps that utilize information from the biological domain, as well as the two transfer steps between the biological and engineering domains. This is because most engineers know little biology or characteristics of animals and plants. A plethora of biomimetic design methods have therefore been proposed to provide this support [9].

### 2.1. Identification, Selection and Abstraction in the Biological Domain

Fayemi et al. [1], and Vattam and Goel [6] investigate how designers may be helped during biomimetics. Assessment of various design methods and tools relevant to biomimetics has shown that these support only one or two steps in the entire biomimetic process [24]. Furthermore, tools can be expected to be helpful in overcoming the challenge of selecting a biological model, and support for the abstraction steps is under-represented [4]. Vattam and Goel found that a much time is wasted by: (1) browsing for possible information sources; (2) searching for relevant data within these sources; and (3) developing an understanding of the contents of such sources. These findings were translated into three challenges that designers usually encounter [7,8]:

- Findability: Keyword-based search is inadequate when the designer can only vaguely describe the information needed. This results in either an extremely large number of results or no results at all. In the former case, many information sources have to be scanned. In the latter case, the query often has to be refined over and over again.
- Recognizability: Poor recognizability of relevant information sources is attributed by Vattam to the lack of biological background knowledge [25]. Time is therefore wasted in identifying potential information sources accurately. The main form of identification is based on term matching and thus semantic similarity, but structural and pragmatic similarities are neglected. Therefore, often, time is wasted on irrelevant information sources, and relevant information sources may be missed.
- Understandability: Typically, designers are unfamiliar with the domain of the information sources. Therefore, the cost for developing a mental model of a potential biological system is high.

Note that findability, recognizability and understandability can be mapped to the identification, selection and abstraction steps in Figure 1.

### 2.2. Biological Information in the Language of Engineers

Vattam and Goel propose to address the three challenges described above through the semantic annotation of biological information in engineering terms [8]. The resulting terms of functions, structures, mechanisms, physical principles and operating environments are thought to improve findability and recognizability because engineers are forced to focus on decomposing their problem. Additionally, the annotations are noted to ease the understandability of the biological texts [25]. Another tool that encodes biological information in engineering terms is AskNature [26]. This online database contains descriptions of over 1500 biological strategies classified in a functional, hierarchical taxonomy. The use of this tool is claimed to increase the novelty of solutions, while maintaining comparable technical feasibility [27]. Facilitating a better understanding of the underlying principles with illustrations was found to further improve the quality and novelty of ideas. However, the AskNature database is populated by hand. This takes a lot of time and is prone to bias, and using design theories during this process may further increase time and effort [10,20].

The vast amount of available biological knowledge remains however largely untapped [28]. Several biomimetics databases contain fewer than 25 complete entries, and the time to create an entry may take up to 100 h. Providing biological information in terminology that is accessible to engineers requires abstraction of biological systems. This partial solution-driven biomimetics process has to be automated if the inherently limited coverage of biomimetic databases is to be increased. In conclusion, there is a need for scalable methods and algorithms to support engineers in biomimetics [29]; hence, the need for CAB tools.

### 2.3. Information Sources for Computer-Aided Biomimetics Tools

To facilitate a scalable approach that is not limited to known biomimetic solutions, one may draw upon the vast amount of existing biological knowledge captured in biological texts [10]. Biological research papers present up-to-date information. The authors of peer-reviewed papers can be assumed to be experts in their field and can be contacted for advice or cooperation. Moreover, the papers are thought to deal with specific details of biological systems [29].

## 3. The Issues Underlying the Search Problem

### 3.1. Function as a Central Concept in Biomimetics

Function has long been the key concept supporting findability, recognizability and understandability in biomimetics. Biomimetic solutions are sometimes regarded as “novel technologies developed through the transfer of function from biological systems” [30]. Inkermann et al. note how Rechenberg in 1979 proposed identifying a desired function, finding a biological analogy and validating its appropriateness before transferring knowledge [31]. Gramann [32] and Stricker [33] mention the algorithmic search strategy for biological functions elaborated by Zerbst from 1987, where a comparison between analogies is made on the basis of functions, constraints and quality criteria. Hill proposes the search for abstract biological functions through a catalog of 191 biological systems divided over 15 descriptive technical and biological functions [34]. Furthermore, more recent research on biomimetics revolves around engineering functions, e.g., Nagel lists other approaches that use function to capture a biological system or phenomenon [35].

However, Stricker notes that biological information sources often do not describe functions, physical relations or the performance of biological systems, their associated phenomena or underlying principles. In a case study on modeling the jumping behavior of a flea, he found little information in biological texts of interest to an engineer. As a work-around, Stricker based his abstracted model of a jumping flea on electron microscopy images [33]. Section 3.2 elaborates on this lack of engineering information in biological texts, which can impede the identification of relevant information sources.

Helms et al.demonstrate that biological texts that do not contain engineering terminology are still useful to mechanical engineers, even when these engineers lack a biological background [13]. Computational systems, on the other hand, especially those that aim to reason over biological and engineering information or map information across domains, require more formal definitions of terms that may indicate functions. Moreover, a biologist may not define a function to the satisfaction of an engineer, which complicates searches and can lead to misapplication of terminology and wrong or simplified interpretations of biological functions [9,36]. Section 3.3 elaborates on definitions and the use of the term function in engineering, biology and biomimetics. To support the structuring and understanding of biological information in an engineering context, various theoretical models exist, e.g., through functional modeling. Section 3.4 addresses the use of theoretical models to represent biological systems, which can help the abstraction of engineering information from biological texts.

### 3.2. Function Used as Keywords as a Tool for Findability

To bridge the linguistic gap between biology and engineering, natural language approaches to biomimetics usually search biological texts for words associated with engineering [20,21,37]. Kaiser et al. evaluate whether it is reasonable to use functions in a search, since functions are not the only descriptors of a system [10]. They investigate additional descriptors as design characteristics and environmental interactions. The latter may be useful during identification because they are not unique to any one system and so can be formulated more globally. Furthermore, biological systems of interest might be filtered based on these constraints. Table 1 provides an overview of search-term categories and respective findings.

Biological research papers can be retrieved not only by terms describing technical functions, but also terms describing properties of technical systems or their environment [10]. Kaiser et al. investigate which type of search terms are most efficient and most effective for searching biological research articles in a biomimetics process [12]. The following types of search terms are examined [13]:

- Function: the intended input/output relationship of a system whose purpose is to perform a task; this definition is based on Pahl and Beitz [39].
- Property: internal properties and purpose properties defined by Eder and Hosnedl, where a property is anything owned/possessed by a technical system [40].
- Environment: all environmental effects on a technical system, as well as the interplay between a system and its environment.

Findings include that search terms from any of the above categories can be effective, combining terms from different categories improves search results, and which terms to use depends on the problem [12]. The efficiency of search terms describing functions was found to be only 20%, which, according to Kaiser et al., can be attributed to the generality of engineering functions. In case functional information does not suffice for the search, Vattam and Goel found that environmental information can be used [41]. Such environmental terms often co-occur with functional terms, suggesting that the capacity of an organism to perform a function might be strongly aligned with environmental influences [12]. In line with this suggestion, adding environmental terms to a search was found to increase the precision of retrieved results in general. Notably, none of the investigated 115 articles contained functional search terms alone.

Based on these findings, we claim that keywords in the category function cannot be expected to always bridge the terminological gap between biology and engineering. Indeed, a biological paper that turns out to be a significant source of inspiration may not even refer to an engineering function. This reduces the usefulness of systems that look for words associated with functions in biological texts, with the aim to instantiate functional models or annotate these texts with engineering terminology. On the other hand, environmental characteristics can provide additional information about a biological system, and biological properties may be used to specify a problem at a lower level of abstraction [12].

### 3.3. Functional Analysis as a Tool for Recognizability

Artefacts and biological systems may both be explained in terms of function. However, Fayemi et al. argue modeling a biological system in terms of parts that are associated with functions is considerably harder than modeling a technical system. This is because the sparse notions of function in biology research papers cannot be assumed to express the same meaning as engineering functions [14]. Reliable recognition of cross-domain terminology depends on a unified definition of the core concepts [36]. However, for the core concept of function, a unified definition that works well for both biological and engineering systems does not exist [42].

Instead of focusing on a unified definition of function, Perlman proposes to acknowledge the advantages various views of function provide in adequately explaining different phenomena [43]. Regardless of its precise definition, function is seen as a useful tool to indicate and clarify relations between physical entities and the interactions of these entities with the environment [36]. Thus, functional capacity is linked directly to environmental influences, as noted in the previous section. The systemic environment then plays a crucial role in understanding a function [43].

Components of a system can be described within the context of the system at various levels of abstraction. Depending on the contextual variables taken into account, the same component can fulfil different functions. An example is a fish’s swim bladder. It provides buoyancy in the water column. At a higher level of abstraction, the swim bladder introduces a lumen, and is therefore a cellular structure. The function of this lumen in some organisms is the improvement of precision in sensing water pressure [44]. The swim bladder is also in some cases used to aid sound production and hearing [45]. When analyzing a swim bladder as a model for the control of buoyancy, one has to consider that certain properties of the system’s components may not be optimised for the function of interest or not contributing to this function at all. Helms et al. give the example of a group of designers that studied the structure of moss with the goal of surface area optimisation for the gathering of sunlight; they took no account of the organism’s requirements for conserving water and for protection [15].

The interrelations between components of biological subsystems can be highly complex. Owing to the inherent multi-functionality of most biological systems “a direct transfer of a biological system into a technical system is rarely possible and often does not make any sense” [31]. One could argue that a biological system can have several functions, and if these functions and the involved systemic components are properly recorded, the assignment of a single function to a single biological subsystem is still valid. A computational tool might then encode the systemic components and their interactions, as well as the influence of the various roles a single systemic component performs in achieving different functionalities. The question then becomes whether it is possible, or desirable, to encode all this information automatically.

We claim that automatically extracting engineering information from biological texts is undesirable. Reliable mapping between functional properties found in biological research papers and engineering applications requires formal definitions of terminology in both domains. Instead, we propose the use of a computational tool that extracts biological information from biological sources. Mapping from biology to engineering and making decisions on the relevance of information on the systemic context have then to be done by a person. This aim is different from the work by Nagel and Stone, where a thesaurus provides term mapping between engineering and biology [23]. While a thesaurus enables keyword expansion across domains, the thesaurus does not scale easily to cover more conceptual similarities.

Extracting within-domain information enables a CAB tool to follow a pragmatic teleo-pluralist approach to function [43] and extract relations between systemic components. This approach takes into account contextual variables, such as environmental interactions, that influence functional capacity. Alternative approaches to the categorisation of biological texts, such as grouping by morphological or behavioral aspects, may further help understand interrelations in biological knowledge [46]. Computer-Aided Biomimetics tools that apply natural language analysis may then use verbs as indicators of both passive and active relations between systemic components, the latter often denoting an intent or behavior independent of context.

### 3.4. Modeling Natural Systems as a Tool for Understandability

In biomimetics, the discrepancies between the concept of design in biology and in technology can lead to wrong interpretations of the origin of biological structures and function [47]. An option would be to regard a function as a role within a context, which allows for notions of functionality that do not assume intention [48]. This is a mode of thinking habitual in biologists.

Understanding the roles that various components have in a system eases the abstraction of relevant information. There is a plethora of biomimetics methods that aim to support understanding and transfer of abstracted knowledge from biology to engineering. Often, these methods model biological analogies in terms of engineering functions. However, Helms et al. found that complex functions are often oversimplified in the analysis of biological systems [15]. Rich, multimodal representations of biological systems, at different levels of abstraction, help avoid oversimplification during analysis [16]. These representations should capture both functions and their mechanisms, on the one hand, and affordances and constraints, on the other.

Representations of biological systems are key to knowledge transfer in biomimetics according to Sartori et al. [49]. Their definition of knowledge transfer is “the reproduction of information from a model of a biological system in a model or prototype for a technical system” [50]. To support the transfer of a biological process, they propose a model comprising State-changes, Actions, Parts, Phenomena, Inputs, oRgans and Effects (SAPPhIRE). This model represents causality in natural and technical systems at different levels of abstraction [51]. In a comparison of a variety of modeling methods, Durand et al. found that all these methods proved to have certain benefits. They conclude that, although differences exist, any modeling methods can be effective and help in generating final concepts [52].

Furthermore, representing a biological system at different levels of abstraction is generally found to be beneficial [9]. Gramann for example proposes an associative check list to support transfer of biological functions to technical functions, aiming to support analysis and synthesis in a typical system engineering procedure [32]. If a technical analogy cannot be adduced from a biological system, one is advised to reconsider the level of abstraction. If the level of abstraction is thought to be adequate, one should then reconsider the assumed goal of the biomimetic study [53]. In a sense, this method proposes iteration over various abstraction levels and alternation between defining the problem and generating solutions.

Creating multiple rich, multimodal representations at different levels of abstraction is a way of rationalizing ideas and developing understanding. The process of designing and developing design understandings has been called distributed cognition [54]. Instead of prescribing one single model, biomimetics may benefit from using a variety of abstract representations that are essential to guiding the thought process. That is, existing modeling methods such as functional modeling [55], Structure–Behavior–Function (SBF) models [21], SAPPhIRE models [50] and the model proposed by Fayemi and colleagues [14,56] may be used side-by-side to shed light on different aspects of a biological system.

## 4. A Holistic, Iterative Approach to Generate Compound Solutions

Knowledge, and thus some analysis and understanding, is required for successful abstraction, probably combined with further investigation [11]. However, for someone who knows little biology, it will be difficult to solve problems in biomimetics through a classical, logical, sequential approach. An iterative, flexible approach, expanding upon various types of models, affords revision and development. Such a flexible approach helps to deal with the interdependencies that exist between available knowledge, abstraction and transfer.

**Figure 2:**
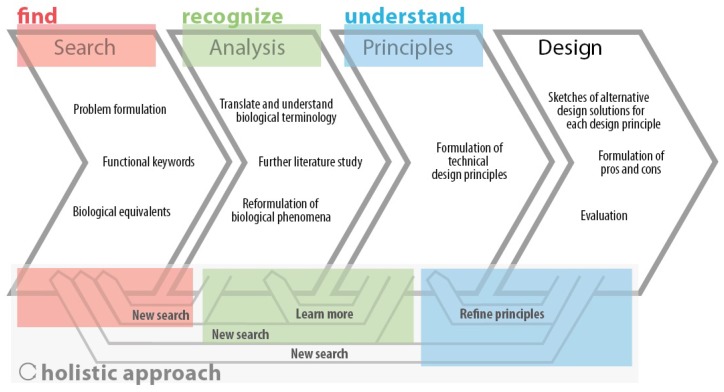
Holistic approach to biomimetics, through supporting identification, selection and analysis by addressing the findability, recognizability and understandability of information. Design process adapted from Lenau [58].

A holistic approach integrates local and global views of knowledge, supporting analysis, the synthetic ability to see problems in new ways and supporting creativity [57]. This enables iterative: (1) analysis of an engineering problem; (2) transposition to the biological domain; and (3) transposition back to the specific engineering setting. Importantly, this approach accommodates an initial lack of biological knowledge. Figure 2 displays how supporting findability, recognizability and understandability can contribute directly to a holistic approach. Previous iterations improve search queries, the acquisition of relevant background knowledge and refinement of abstractions. Previous iterations help also to focus on the most probable solution routes, compound multiple analogies and the co-evolution of problems and solutions [18]. It comes as no surprise that Helfman Cohen and Reich define abstraction as “the process of refining the biological knowledge (design solutions) to some working principles, strategies or representative models that explain the biological solution and could be further transferred to the target application” [11].

Schön argues our knowing is in our action and interaction, which relates to the rationalisation of a thought process by externalizing ideas [17]. That is, representing your current knowledge on, e.g., a biological, technical or biomimetic system provides additional insight into the problem and possible solutions. Fayemi has tried to validate the benefits of existing biomimetic design tools through case studies with multidisciplinary teams, using feedback and statistical methods to demonstrate the efficacy of the methods [56]. The need is for a scalable and reliable method to provide relevant biological knowledge during biomimetic problem-solving. Table 2 provides an overview of the challenges CAB tools should aim to overcome.

## 5. Related Computer-Aided Biomimetics Research

This section briefly describes related research on CAB tools and the type of information they aim to provide to engineers. An implication of working with biological research papers is that CAB involves text processing. Therefore, the scope is limited to CAB tools that apply Natural Language Processing (NLP).

### 5.1. Searching through Natural Language for Relevant Biological Words

Vakili and Shu searched through an introductory biology textbook [37]. This method cannot be expected to be used directly with other types of biological texts, e.g., research papers [29]. The search keywords used are verbs that describe the desired effects of a possible solution, as well as words that are often found in excerpts of text together with the target verb, so-called biologically meaningful keywords [59,60,61]. The NLP approach was found in general to retrieve either nothing, or many irrelevant results [19,62].

To improve the recognizability of search results, functional modeling has been explored [23,63]. This uses the functional basis, defined as a taxonomy for standardised function-related terminology in verb–object format. This taxonomy is built on the assumption that it is possible to identify a comprehensive, minimal set of nonoverlapping functions that can be used to model engineering artefacts, products and systems [64]. By mapping biologically meaningful keywords to verbs from the functional basis, Cheong et al. search for relevant biological text excerpts using engineering functions [38]. They find that the recognizability and understandability of search results in a NLP approach can be improved through clustering and relating terms [65].

### 5.2. Semantic Similarity between Biological and Engineering Concepts

The Scalable Search for systematic Biologically Inspired Design (SEABIRD) system clusters biological terms as described in Table 3 [20,66]. Because function and environment are strongly aligned, semantic concepts such as Organism Aspects (OAs) can capture related terms at several abstraction levels. However, a technique such as latent semantic indexing does not cope with synonymy and polysemy. For example, a polysemous noun such as ’vessel’ can introduce noise by linking some OA representing a circulatory system to a Product Aspect (PA) representing flotation devices. More sophisticated NLP techniques may be more reliable, e.g., by introducing word sense disambiguation, and thus, increase the relevance of retrieved systems.

Using different methods, both Vandevenne et al. [20] and Cheong et al. [38] map engineering terms onto biological terms based on the co-occurrence of words. This does not provide insight into the relations between terms, but Vandevenne et al. note that other research could be integrated to provide such support in the SEABIRD system. Amongst others, extracting causally related functions and automatic instantiation of SBF models are mentioned [70,71]. The former help understand how one function is enabled by another. The latter represent relations such as causal mechanisms and interactions between system components.

### 5.3. Relating Terms and Modeling System Component Interactions

The Intelligent Biologically Inspired Design (IBID) system automatically extracts SBF models from biological research papers; also, see Table 4. Rugaber et al. assume that functional modeling can robustly represent mechanisms in both engineering and biology. However, this does not imply that functional representations can be automatically extracted from biological research papers. The extracted models were found to be correct, but incomplete, because the sub-models are not fully interconnected. “For example, some of the structural elements it extracts do not appear to play any role in the accomplishment of the system functions” [21].

Kim and Lee recognize that function-based representations of biological systems do not provide the whole story, which negatively affects the retrieval of relevant systems. In order to overcome the usual bias of biomimetic modeling methods towards physical relations, they propose to use the biological characteristics described in Table 5. The resulting bionic Metadata Information Research retrieval system outperforms AskNature in an empirical evaluation of the precision of search. The system indexes biological systems found in the AskNature database using an extended SAPPhIRE model. Although the descriptions of biological systems from AskNature are automatically transformed into the extended SAPPhIRE format, no additional models of biological systems are instantiated [74]. Regarding the scalability of the system’s database, the use of a detailed theoretical model can be expected to complicate the automated population of a database [28]. Moreover, the resulting models do not always facilitate a better understanding of biological systems in comparison with purely textual or diagrammatic representations [16].

## 6. Proposed Computer-Aided Biomimetics Support

This section introduces an approach to biomimetics that enables the use of a CAB tool with the characteristics listed in the Introduction. The aim is to: (1) describe how a CAB tool, which avoids pre-emptive cross-domain transformations of text sources, can provide the support outlined in Table 2; and (2) illustrate the resulting biomimetic design process.

### 6.1. Identifying Trade-Offs

**Figure 3:**
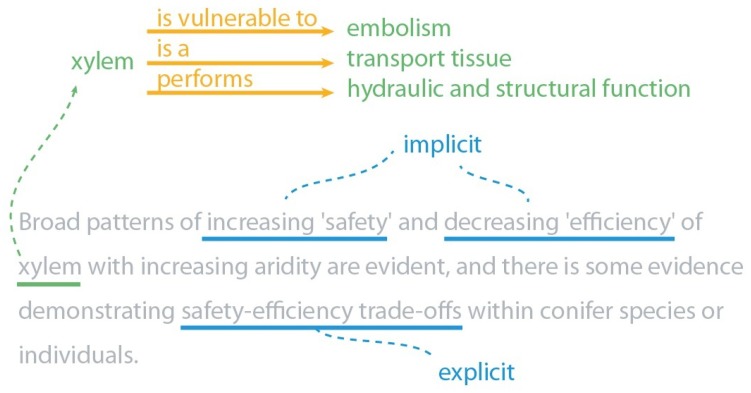
Example of a sentence that contains both an implicit and an explicit trade-off [77]. Automated extraction of trade-offs may take advantage of both. Furthermore, extracting information on concepts and relations in the text can provide a summary view, e.g., a term like xylem may be unknown to an engineer, but relations found in the text may quickly clarify its meaning and properties. A system may further clarify such biological concepts using information from other sources or, e.g., add short descriptions from a dictionary.

The trade-off is a central concept in biology, strongly associated with speciation and adaptation; a driver for change. It denotes that a certain trait cannot increase without a decrease in some other trait [76]. An example is the common trade-off between speed and accuracy. In general, a process is less accurate when it is performed faster, and can be more accurate when performed slower. As visualised in Figure 3, trade-offs are present both implicitly and explicitly in biological texts. Like man-made systems, biological systems optimize and compromise between such conflicting goals. Vincent describes how trade-offs between high-level concepts provide a bridge between biology and engineering, enabling the classification of the conflicting objectives that a system has to balance [2]. Currently, this classification is done by hand and only partially supported by natural language analysis.

### 6.2. Recognizability

**Figure 4:**
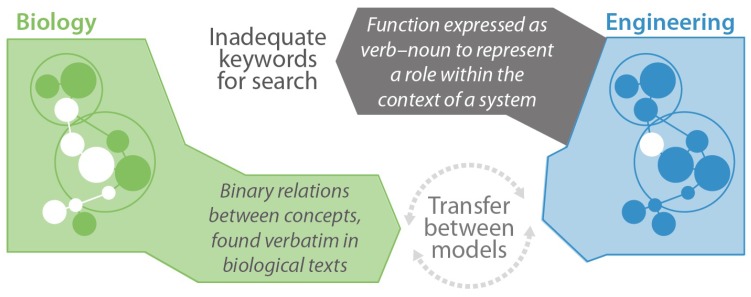
Rather than searching for biological research papers using engineering terminology (top: function bridge), the proposed approach aims to support the modeling of biological systems through elucidating the concepts and relations in a text (bottom: modeling context of a process).

Trade-offs mentioned in biological research papers can provide an initial filter for the identification of relevant information sources. Further filtering can be expected to be necessary, preferably in a manner that requires little biological domain knowledge. Clustering and relating biological concepts are expected to play a significant role in the support of identifying, recognizing and understanding relevant biological analogies [25]. Specifically, the relations are key to successful analogical transfer, because analogical reasoning revolves around higher-level relational similarities [67]. Figure 4 illustrates how the function bridge between biology and engineering is replaced by iterative modeling of information from both domains. Elucidating important concepts and relations can support modeling of biological systems and thus support knowledge transfer between domains.

### 6.3. Understandability

**Figure 5:**
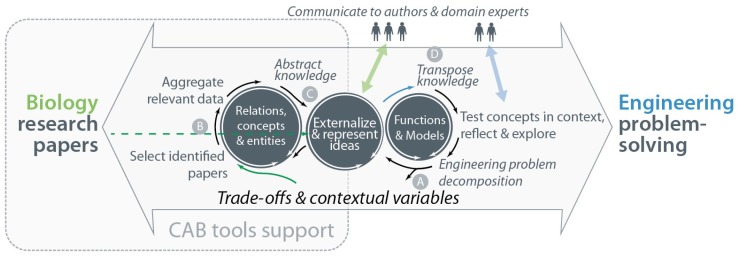
Overview of the holistic, iterative approach that may be supported by a Computer-Aided Biomimetics (CAB) tool. Steps may follow each other in nondeterministic order. One possible route through a CAB: (A) Define an engineering problem as a trade-off, enabling an initial search for biological research papers; (B) Select several sources of data, and aggregate the information that seems relevant; (C) Abstract relevant knowledge from the information sources, and represent this separately from the problem context; (D) Validate the represented ideas and transpose the knowledge to the engineering problem, enabling the implementation and testing of the conceptual solution.

Contextual variables, e.g., environmental characteristics and properties, may be linked through trade-off relations, causality, correlations, associations, separations, processes and identities [2]. Words representing these variables provide keywords to filter and search for information. By mapping and relating the contextual variables that occur in various papers, large amounts of information may be integrated in the design process. To accommodate the iterative character of such a process, holistic design is proposed as visualised in Figure 5. The aim is to enable compounding of knowledge at different levels of abstraction, a crucial feature to support successful biomimetic knowledge transfer [50]. The use of this approach is explained further using a case study.

The process in Figure 5 illustrates a continuous challenge between defining the problem and generating one or more solutions. This iterative process incrementally leads to a richer representation of biological and technical models. New directions for solutions may be explored when stumbling upon interesting properties of analogous biological systems [78]. The exploration, experimentation and communication of raw ideas throughout a design process helps to rationalize thought [79]. New insights and ideas are manifested through a continuous interaction with predetermined or loosely defined constraints, reflection-in-action, reflection-on-action, reflection-in-practice and communication [17,80]. The systematic gathering of relevant biological knowledge then enables a more specific search.

### 6.4. Example of Process

This section illustrates how the proposed biomimetic process could unfold, as indicated by Steps A–D in Figure 5.

**Step A: Define the engineering problem.** Based on an engineering problem, a user is expected to define one or more trade-offs that then define the solution space. One might search for several trade-offs and then identify a controlling trade-off. As an example, the accuracy and speed of movement of an industrial robotic arm influence processing time and cost. A biological system may provide insight into methods to improve the repeatability and accuracy of a robotic arm that has to perform actions quickly.

Vincent [2] gives several examples of biological systems that operate in the domain of a speed-accuracy trade-off; a brief overview of the first two biological systems is given in Table 6. One might start investigating a paper that describes the capture of prey by the antlion larvae [81]. A second example of a biological system that may initially seem less relevant is the hunting behavior of an archer fish [82]. For the purpose of this example, we further explore how the archer fish might inspire a solution.

**Step B: Support the selection of further papers.** The initial information source does not describe how the archer fish can align its body with the estimated falling location of a prey so rapidly [82]. It does, however, show that the fish bases its prediction of where the prey will fall using less information than a normal trigonometrical construction requires. Thus, it cuts down on information to be processed and saves time. Notably, we assume that initially identified information sources are unlikely to contain all information to solve the engineering problem. The reason is that biological research often does not fully explain the biology, but aims to describe one or more specific properties of a biological system. Through iteratively searching for further information, a more complete understanding of the entire system is compounded.

Selecting further information sources can be based on the references that accompany extracted relations of interest; e.g., Berry et al. are cited, as the processing of visual cues may be expected to take 30–100 ms, emphasizing the extraordinary quickness of the archer fish’s reaction [83]. Furthermore, various publications by one author may provide additional information on the same topic:

- Schuster et al. show that archer fish learn to estimate absolute sizes of aerial objects despite complex optical distortions from their underwater viewpoint. Notably, the fish is thought to learn a concept of objective size instead of interpolating estimations using past experiences [84].
- Schuster et al. describe that the fish can learn to hit targets that move in three-dimensional space, by training them with targets that move in only one direction. They can even learn this skill by watching another archer fish hunt [85].
- Wöhl and Schuster find that the fast and precise predictive alignment manoeuvre shows all hallmarks of Mauthner-driven teleost C-type fast-starts [86]. C-starts are very fast escape reflexes that start with the fish bending its body in a C-shape. Many fish and amphibians can perform this C-start escape. The predictive fast-starts that archer fish perform are among the fastest known C-starts and do not perform slower than archer fish escape C-starts. The kinematic equivalence and identical temporal pattern of both starts suggest that the neural C-start escape network is used to drive the predictive starts.
- According to Schlegel and Schuster, the computations that underlie the predictive C-start must be done in the retina and the fastest output pathway, making it likely that a small network of six identified neurons plays a key role [87].

The decision of a user to spend time, and the time spent, on an information source may be considerably sped up by an information extraction tool; also, see Section 6.5. Based on the information in the set of six papers describing the archer fish, one can form an initial idea of how this fish is able to balance speed and accuracy during a predictive C-start. If one decides to pursue this as a solution direction, a more directed search is possible; e.g., adding Mauthner cells as an additional search concept. Notably, spending a short amount of time on a paper that turns out to be irrelevant to solving the problem may still be helpful in the selection of information sources later on.

**Figure 6:**
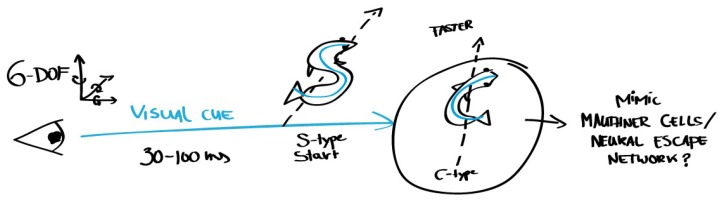
Example of simple sketches to capture abstracted relevant knowledge from several information sources, in this case those listed in Step B. Such a simple sketch can help validate design ideas, e.g., point a designer to re-examine [86] for more information on S-starts and C-starts. Alternative modeling methods may be used to capture specific types of information, e.g., by following existing biomimetic transfer models.

**Step C: Abstract knowledge.** Another aim of the CAB tool is to help recognition of relevant information by automatically unveiling relations and concepts mentioned in the text. Understanding of a biological process or system in its context is achieved through the representation of information gathered throughout various papers. Such representations could take the form of, e.g., sketches as in Figure 6, notes and instances of existing knowledge transfer models. The incremental expansion and revision of the user’s knowledge supports search, selection and abstraction.

**Step D: Transpose knowledge.** It is up to the user to validate the abstracted understandings and judge whether transposition of the knowledge to engineering is feasible [23]. Further research on a fish’s neural escape network may provide the basis for a conceptual solution to the example problem in this section, which might be tested and implemented in the engineering context. This step represents the actual knowledge transfer between the engineering and biology domain. The CAB tool would not necessarily provide any support here. Rather, information processing across domains is avoided in favour of a more reliable support for the speedy identification, selection and abstraction of relevant information.

### 6.5. Towards Information Extraction

To take advantage of a trade-off bridge, NLP tools are required that automatically extract trade-off relations from biological research papers and annotate them with further information for additional filtering. To this end research on the proposed CAB tool focuses on information extraction; the process of automatically extracting structured information from unstructured or semistructured documents [88]. Among the types of information that may be extracted are the central concepts that appear in a text, such as entities, relations between entities and attributes describing entities [89]. The extracted information may be stored in a Knowledge Base (KB), enabling computation over annotated, unstructured information sources. Tasks that benefit from the use of such KBs include information retrieval, reasoning and question answering [90].

To investigate how trade-offs between high-level concepts are expressed in scientific papers, a set of 200 papers was analysed manually. Subjects ranged from biomechanics to ecology and cellular mechanisms. The papers were selected to deal with a problem and providing a solution. Purely descriptive papers and those reporting results with little analysis were not selected. Eighty-five papers were found to solve a stated problem with reasons. Fifty-nine of these papers were identified as describing a trade-off, but only 25 explicitly mentioned trade-off verbatim. Alternative terms included compromise, optimisation, balance, safety factor, antagonism, interplay, conflict and incompatibility. The range and variance of expressions used to indicate a trade-off, both explicitly and implicitly, limits the coverage of a keyword or rule-based approach to find trade-offs in text. Statistical and machine learning techniques deal better with noise and generalize better than the rule-based approaches currently applied in CAB systems [89].

## 7. Conclusions

Problem-driven biomimetics remains largely adventitious due to the lack of a suitable bridge between biology and engineering. To improve the chances of finding out-of-the-box solutions from biology, computational tools may enable the integration of large amounts of biological knowledge. The set of all biological research papers is assumed to represent comprehensively the biological domain. Several existing CAB tools apply natural language processing techniques to identify the biology research papers relevant to a given engineering problem. However, these tools attempt to map information between the biological and engineering domains. This mapping is unreliable due to the semantic differences between biology and engineering terminology.

Instead of focusing primarily on information retrieval, we suggest to focus on within-domain information extraction initially. While functional relations can be useful to indicate the interaction between physical entities and their environment, engineering functions cannot be assumed to be found verbatim in biological texts. The automated annotation of biology research papers with engineering terminology can therefore obscure unreliable extractions. This considerably reduces the usefulness of automatically instantiated models for knowledge transfer.

Trade-offs provide a bridge between biology and engineering. Trade-offs are a central concept in biology and indicate a dialectical relationship in abstract concepts. To overcome the lack of affordance for accurately perceiving the information content, biological research papers should be classified automatically and trade-offs annotated. Semantic concepts that capture contextual variables, such as operating environment and biological materials and structures, can filter the results further.

This paper presents a pragmatic approach to biomimetics that takes into account the differences between biological and engineering systems, as well as the lack of biological knowledge among engineers. A limitation of this study is the strong focus on developing computational tools to support biomimetics. While scalability and integration of knowledge are primary goals, they affect the ease of use in comparison to, e.g., databases, where some form of preliminary analysis has been performed.

The approach presented in this paper provides a basic conceptual framework for developing CAB tools. Current research focuses on the information extraction prescribed by this framework. We have investigated relation extraction from scientific publications and are currently preparing a dataset to train information extraction systems for CAB.

## Figures and Tables

**Table 1:** Categories of terms used to annotate predetermined pairs of biological and technical texts. For each pair, the subject of the biological text could possibly result in a biomimetic solution for the chosen technical context. The findings are based on the analysis of four pairs. Based on Kaiser et al. [10].

Search-Term Categories	Findings
**Function** Functional verb that, together with an object, constitutes a function of the particular system (e.g., transport water)	Kaiser et al. show that functional verbs cannot always be found. In case functional verbs are found, they can be matched without WordNet expansion [10]. Biologically meaningful keywords by Cheong et al. do not add any matches [38].
**Function owner characteristics** Characteristics of a particular system’s function owner	Terms in this category were not always found. If present, WordNet expansions, including antonyms for identified synonyms, and matching across categories can lead to additional matches.
**Function owner** Carries out the considered function of the particular system	Often described in discipline-specific terms and therefore not considered for matching.
**Interacting conditions** Environmental interactions with the particular system	Terms in this category were found in all four described cases. Furthermore, WordNet expansion allowed for successfully matching the biological terms to technical terms.

**Table 2:** Overview of the challenges Computer-Aided Biomimetics tools should overcome and the related themes of common mistakes and recurring findings. Adapted from Kruiper et al. [9], and Vattam and Goel [6,7].

Themes	Challenges
**Scalability**	Ability to integrate large amounts of biological knowledge to support biomimetics.
**Formalisation** **Transfer impediments** **Validation** **Analogies**	**Findability–Identification** Inadequacy of keyword-based search **–** Huge amount of results or no results at all
**Transfer impediments** **Validation** **Analogies**	**Recognizability–Selection** Lack of domain knowledge, leading to a focus on semantic similarity while neglecting structural and pragmatic similarity **–** Time wasted identifying potential information sources **–** Time wasted investing in irrelevant information **–** Time wasted overlooking relevant information sources
**Transfer impediments** **Validation** **Abstraction**	**Understandability–Abstraction** Lack of domain knowledge, resulting in a high-cost for developing a mental model of potential biological systems
**Holistic Approach**	Ability to alternate between problem decomposition and analogical reasoning, simultaneously expanding the designer’s knowledge required for validation.

**Table 3:** The Scalable Search for systematic Biologically Inspired Design (SEABIRD) system and Product Aspects in Design by Analogy (PAnDA) tool cluster sub-models are used as indices when searching a repository of processed and annotated biological research papers. They are independently extracted from biology research papers and linked. Adapted from Verhaegen and colleagues [67,68] and Vandevenne et al. [20].

Organism Aspects (OA)	Product Aspects (PA)
300 Manually labeled clusters, representing the nouns, adjectives, verbs and adverbs found from 8011 biological research papers. These parts of speech are assumed to represent biological functions, properties and environmental terms.	300 Manually labeled clusters, representing the nouns, adjectives, verbs and adverbs found in a corpus of 155,000 technical patents. These represent engineering functions, properties, technologies and application domains.
Both the SEABIRD and PAnDA system extract information on the basis of term occurrences in text, in a way that is noted to be mathematically close to latent semantic indexing. Hence, when adding a significant number of new documents to the system, further filtering and grouping of terms will be required. Cross-domain linking of OAs and PAs provides a conceptual abstraction level that is central to the approach (Vandevenne et al. [69]). The links are established through a similarity computation based on the overlap in words that comprise the OAs and PAs.	

**Table 4:** The Structure–Behavior–Function (SBF) sub-models are used as indices when searching a repository of processed and annotated biological research papers. They are independently extracted from the papers and interlinked. Adapted from Goel et al. [71], Spiliopoulou et al. [72], and Rugaber et al. [21].

SBF Sub-Models	Method of Extraction
**Structure** Physical components and the connections amongst them	To find structures in biological texts, a vocabulary of biology-specific structures is used, containing over 200 structural components and connections. This vocabulary is based on an ontology for biomimetics by Vincent [2,73].
**Behavior** Description of causal mechanisms	Behaviors arise from the interactions among structural components and contribute to the system’s functions. To determine causality, syntactic patterns are used, similar to those used by Cheong and Shu [70].
**Function** Abstraction of the system’s actions on its external environment	The function sub-model is extracted using a domain-independent controlled vocabulary, based on the functional basis and the biomimicry taxonomy of AskNature.

**Table 5:** To comprehensively capture the various relations found between system components, both internally and externally, these biological characteristics extend the usual function-based modeling approach. Adapted from Kim and Lee [74].

Type of Relation	How the Relation Is Captured
**Physical relations** Physiological features	Resource usage and flow that affect physiological features of organisms; as an example, the phenomenon “absorb moisture” may be achieved through the effect of capillary action. These relations can be expressed using SAPPhIRE models.
**Ecological relations** Geographic, ecological and behavioral features	Ecological phenomena and behavior; as an example, “avoid predator” is achieved by camouflage. The distinction from the physical relations is that these interactions take place on an ecosystem scale and may not be recognized directly as engineering functions. To capture these relations, the SAPPhIRE model is extended to cover ecological phenomena and effects.
**Biological relations** Morphological and molecular features	So-called identifying keys from the DEscription Language for TAxonomy (DELTA) system [75], used in phylogenetic classification, are used to find organisms that have similar features. The relatedness between the species is assumed to capture biological relations. More than 21,000 related species are linked to database entries.

**Table 6:** Overview of two possibly relevant biological systems within the solution space of a speed-accuracy trade-off. The information listed as possibly relevant has been manually extracted from the information sources by quickly scanning through the documents. Adapted from Lambert et al. [81], and Rossel et al. [82].

Biological Systems	Possibly Relevant Information
**Antlion larvae** A pit-building larvae that uses its mandibles for catching prey	This organism essentially modulates independent bilateral control over a single rotational degree of freedom. Recreating this behavior may be possible through further investigating the antlion’s control system, possibly in combination with reducing the degrees of kinematic freedom. Strikes prey in 17.60 ± 2.92 ms Near-simultaneous contact of both mandibles Modulation of angular velocity based on prey location Trade-off between: Modulate accuracy of the strike Velocity of the strike
**Archer fish** A fish that preys on land-based insects by shooting a jet of water from its mouth	This fish shoots down prey above the water surface and aligns itself with the location where it predicts the prey will land within 100 ms [82]. After alignment, the fish rapidly accelerates to catch the prey before other predators do. The alignment itself is based on a three-dimensional prediction task, taking into account: Speed Direction Target distance
